# Metaciclogénesis de *Trypanosoma cruzi* en *Belminus ferroae* (Reduviidae: Triatominae) y capacidad infectiva de las heces en condiciones de laboratorio

**DOI:** 10.7705/biomedica.5394

**Published:** 2020-10-16

**Authors:** Maritza Alarcón, Cesare Colasante, Sonia Araújo, Reinaldo Gutiérrez-Marín, Dalmiro Cazorla-Perfetti, Claudia Magaly Sandoval-Ramírez

**Affiliations:** 1 Laboratorio de Parasitología Experimental, Departamento de Biología, Facultad de Ciencias, Universidad de Los Andes, Mérida, Venezuela; 2 Laboratorio de Fisiología de la Conducta, Facultad de Medicina, Universidad de Los Andes, Mérida, Venezuela; 3 Grupo de Investigación GIEPATI, Universidad de Pamplona, Pamplona, Colombia; 4 Laboratorio de Entomología, Parasitología y Medicina Tropical, Centro de Investigaciones Biomédicas, Decanato de Investigaciones, Universidad Nacional Experimental “Francisco de Miranda”, Coro, Venezuela; 5 Grupo de Investigaciones en Ciencias Básicas y Aplicadas para la Sostenibilidad, Facultad de Ciencias Exactas, Naturales y Agropecuarias, Universidad de Santander, Bucaramanga, Colombia

**Keywords:** *Trypanosoma cruzi*, Triatominae, enfermedad de Chagas, tripanosomiasis, *Trypanosoma cruzi*, Triatominae, Chagas disease, trypanosomiasis

## Abstract

**Introducción:**

*Belminus ferroae* es un triatomino de comportamiento entomófago, sin embargo, puede alimentarse de vertebrados ocasionalmente. No se ha demostrado infección natural por *Trypanosoma cruzi* en esta especie, como tampoco la metaciclogénesis del parásito.

**Objetivo:**

Examinar la metaciclogénesis de *T. cruzi* en *B. ferroae* y la capacidad infectiva de las heces o sus contenidos intestinales en roedores.

**Materiales y métodos:**

Se analizaron las heces y la orina expulsadas espontáneamente por los insectos o mediante compresión abdominal o extracción del contenido intestinal a los 10, 20, 30, 40, 50 y 60 días. Se cuantificó la carga parasitaria de *T. cruzi* y sus formas evolutivas se identificaron con tinción de Giemsa. Asimismo, se evaluó en ratones albinos la capacidad infectiva de los tripomastigotes metacíclicos de *T. cruzi* obtenidos de las heces o contenidos intestinales de los especímenes infectados.

**Resultados:**

El análisis parasitológico reveló tres (15%) insectos infectados con *T. cruzi* a los 30 (n=1), 40 (n=1) y 50 (n=1) días después de la infección con cargas parasitarias de hasta 1,62 x 10^5^ tripanosomas/mm^3^ y porcentajes de metaciclogénesis entre el 3,5 y el 6,78%.

**Conclusiones:**

Se demuestra por primera vez, en una especie del género *Belminus*. la metaciclogenésis de *T. cruzi* en condiciones de laboratorio y la capacidad infectiva de las heces para un huésped vertebrado.

## Abstract (en)

**Introduction:**

*Belminus ferroae* is a triatominae with entomophagous behavior. However, it may occasionally feed on vertebrates. Currently, there is no evidence of natural infection with *Trypanosoma cruzi* or the occurrence of metacyclogenesis in this species.

**Objective:**

To test *T. cruzi* metacyclogenesis in *B. ferroae* and the infectivity of their feces or intestinal contents in rodents under laboratory conditions.

**Materials and methods:**

Twenty nymphs of *B. ferroae* were infected with an autochthonous strain of *T. cruzi* (M/HOM/VE/09/P6). Fecal and urine samples were collected from spontaneous droppings or by compressing the bugs’ abdomens and, eventually, by removing their gut contents, and then examined at 10, 20, 30, 40, 50, and 60 days. We quantified *T. cruzi* parasitic load, as well as the evolutionary forms in feces, urine, and intestinal contents by Giemsa staining. Similarly, we evaluated the infectivity of *T. cruzi* metacyclic trypomastigotes in albino mice.

**Results:**

The parasitological analysis showed three insects (15%) infected with *T. cruzi* at 30 (n=1), 40 (n=1), and 50 (n=1) days post-infection. We observed parasitic loads of up to 1.62 x 10^5^ trypanosomes/mm^3^ and metacyclogenesis percentages between 3.5% and 6.78%.

**Conclusions:**

This is the first time that *T. cruzi* metacyclogenesis is reported in a species of the genus *Belminus* under laboratory conditions and the infectivity of *Belminus*’ feces is demonstrated on a vertebrate host.

Los triatominos son un grupo diverso de insectos que se alimenta principalmente de la sangre de vertebrados (1). Su importancia en salud pública radica en que están implicados como vectores en el ciclo de transmisión del parásito *Trypanosoma cruzi,* agente etiológico de la enfermedad de Chagas o tripanosomiasis americana, dolencia que afecta entre 7 y 8 millones de personas y se considera la enfermedad de etiología parasitaria causante de la mayor tasa de decesos (10.000 muertes por año) y con el mayor impacto socioeconómico en Latinoamérica (2).

Desde hace más de un siglo, se ha planteado que los miembros de la subfamilia Triatominae presentan un comportamiento principalmente hematófago y se considera que todas las especies de la subfamilia (alrededor de 150), eventualmente, pueden transmitir *T. cruzi* mediante los principales vectores pertenecientes a las tribus Rhodniini y Triatomini (1,3,4). Sin embargo, cabe destacar la existencia en la subfamilia de variados comportamientos alimentarios, como la cleptohematofagia, la coprofagia, y el canibalismo. En forma experimental se ha logrado, inclusive, que alcancen su ciclo de desarrollo completo ingiriendo solamente hemolinfa (5,6). Recientemente, se ha documentado la fitofagia en experimentos con *Rhodnius prolixus* (7) y, en diversos estudios, se ha señalado que estas estrategias permitirían a algunas especies sobrevivir durante cierto tiempo cuando no disponen de un huésped vertebrado (6-9).

Actualmente, la tribu Bolboderini la integran 13 especies de pequeñas dimensiones agrupadas en cuatro géneros: *Belminus*, *Bolbodera*, *Microtriatoma* y *Parabelminus*, siendo los miembros del primero principalmente entomófagos (4,6,10-12). Este género lo conforman ocho especies, por lo que es el de mayor número de especies de la tribu (>60%) (13).

Como en la mayoría de los taxones de esta tribu, es muy poco lo que se conoce acerca de su bioecología e importancia sanitaria, y se les tiene como un grupo de triatominos con hábitos arbóreos silvestres, algunas veces asociados con bromeliáceas, donde conviven con varios tipos de huéspedes vertebrados (didélfidos, bradipódidos, roedores, lagartos) o insectos (2,3,13). Sin embargo, en varios estudios se ha registrado la incursión y la colonización de domicilios por especies como *B. peruvianus*, *B. corredori*, *B. ferroae* y *B. herreri* (10,11,14).

Es importante destacar que solamente en una de las ocho especies (*B. herreri*) se ha detectado infección natural con *T. cruzi* mediante técnicas de biología molecular, pero no por examen directo (10). Por esta razón, al no contarse con evidencia de formas infectivas metacíclicas en el examen microscópico, existe la necesidad de seguir examinando, en especies del género, la metaciclogénesis de *T. cruzi*, la capacidad infectiva de las heces para mamíferos, la sensibilidad de estas especies frente al flagelado y su capacidad para transmitirlo. Este tipo de estudios es escaso en el género *Belminus* y se requiere para determinar la capacidad vectorial de estos taxones (15), así como para estimar su importancia epidemiológica en la transmisión de la enfermedad de Chagas.

*Belminus ferroae* ha sido la última especie descrita del género y la tribu Bolboderini, además de ser el taxón que más se ha estudiado en este grupo (11). *Belminus ferroae* prefiere alimentarse de hemolinfa de cucarachas, no se la ha hallado infectada naturalmente con *T. cruzi,* y se la ha recolectado en domicilios de comunidades rurales del nororiente colombiano (11). Sin embargo, datos experimentales recolectados en la naturaleza indican que es necesario seguir indagando el potencial de transmisión vectorial de *T. cruzi* (11). En este sentido, se sabe que *B. ferroae* incluye tanto animales como humanos entre sus huéspedes (11). Por otra parte, en una experiencia piloto se evidenció que, en condiciones de laboratorio, sus ninfas e imagos fueron sensibles a la infección por *T. cruzi* (15).

En este contexto, en este estudio se exploró la metaciclogénesis de *T. cruzi* en ninfas de *B. ferroae* con una cepa venezolana del protista, la M/HOM/VE/09/P6, bajo condiciones de laboratorio; asimismo, se evaluó la capacidad infectiva en ratones albinos de tripomastigotes metacíclicos de dicha cepa, presentes en heces y contenidos intestinales de *B. ferroae*.

## Materiales y métodos

### Parásitos

Se utilizó la cepa M/HOM/VE/09/P6 de *T. cruzi* I, aislada de un caso humano por contaminación oral ocurrido en el Estado Vargas, Venezuela (16,17).

### Triatominos

Se utilizaron 20 ninfas de quinto estadio de la especie *B. ferroae* mantenidas en envases de plástico (10 x 7 x 2 cm) con folios de papel absorbente en su interior, a una temperatura de 26 ± 2 °C, humedad relativa >90% y fotoperiodo de 12 horas de luz y 12 horas de oscuridad en una cámara climatizada (Biotronette™). Los insectos se alimentaron de cucarachas (*Blaberus giganteus*) por ingestión de su hemolinfa. La colonia fue iniciada en el año 2005.

### Roedores

Como huéspedes vertebrados experimentales, se utilizaron 15 ratones albinos machos heterocigotos de dos meses de nacidos, de 18 a 20 g de peso, mantenidos en el bioterio experimental (22 ± 20 °C; humedad relativa: 54 ± 5%) con alimento comercial *ad hoc* (Ratarina®) y agua *ad libitum.*

### Xenodiagnósticos

Se utilizaron seis ratones para el xenodiagnóstico, los cuales se infectaron por vía intraperitoneal con 2 x 10^4^ tripomastigotes metacíclicos de la cepa M/HOM/VE/09/P6 de *T. cruzi*. Los tripomastigotos metacíclicos se obtuvieron a partir de heces de 20 ninfas IV de *R. prolixus* con 30 días de infección, siguiendo la metodología empleada por Brener (18).

La parasitemia de los ratones se evaluó día de por medio mediante el examen directo de muestras de sangre periférica. El recuento de tripomastigotes ¿sanguícolas? se hizo en 100 campos microscópicos con 400X ¿(19)? ¿Debería ser la 19?; a los 15 días de la infección, cuando los ratones presentaban aproximadamente 3,5 x 10^5^ parásitos/ml, se procedió a alimentar los especímenes de *B. ferroae* después de diez días de ayuno. Posteriormente, se colocaron dentro de envases de vidrio (18 x 7 x 5 cm) con folios de papel en el fondo, en una cámara de ambientación en las condiciones ya señaladas, pero sin exponerlos a la alimentación con el huésped invertebrado (cucarachas).

A los 10, 20, 30, 40, 50 y 60 días de la ingestión de sangre, se hizo la lectura de los xenodiagnósticos mediante el análisis de 2 a 3 ejemplares cada 10 días y, además, se les ofreció alimentación sobre ratones sanos no infectados con *T. cruzi*.

En los casos en que los insectos defecaron o miccionaron espontáneamente, se recolectó una alícuota de 5 µl de las heces o la orina, y se analizaron bajo microscopio fotónico para determinar la carga parasitaria según la metodología descrita por Brener (18). Con el resto de cada muestra, se hicieron tres extendidos finos sobre láminas y se colorearon con la técnica de Giemsa al 10%.

A los insectos que no expulsaron las heces de manera espontánea, se les practicó compresión abdominal y, cuando no fue posible recolectar la muestra, se procedió a inmovilizar el insecto en frío para remover completamente el tubo digestivo con una jeringa de tuberculina bajo el microscopio estereoscópico y luego homogenizarlo en 100 µl de solución fisiológica. Cada alícuota de 5 µl se examinó al fresco y el volumen restante se coloreó con Giemsa. Las láminas se observaron con un microscopio óptico con objetivo de inmersión (100X), y se contabilizaron las diferentes formas evolutivas de *T. cruzi* (epimastigotes, tripomastigotes, formas en transición, formas en división) en 200 campos (20); las fotografías fueron tomadas con una cámara Panasonic de 16 megapixeles.

### Capacidad infectiva de ratones con macerados intestinales y heces de Belminus ferroae

En tres alícuotas de 5 µl de los diferentes macerados intestinales obtenidos, se contabilizó la carga parasitaria o inóculo (4,75 x 10^4^) y, después, se inyectaron a tres ratones por vía intraperitoneal. Posteriormente, se evaluó su capacidad infectiva cada tres días durante 39 días y, en caso de ser positivos para *T. cruzi* (M/HOM/VE/09/P6), se determinó la parasitemia.

### Consideraciones éticas

Los procedimientos utilizados en los xenodiagnósticos y la infección de ratones albinos, fueron avalados por el comité de bioética de la Facultad de Ciencias de la Universidad de Los Andes, Mérida, Venezuela.

## Resultados

**Figura 1:**
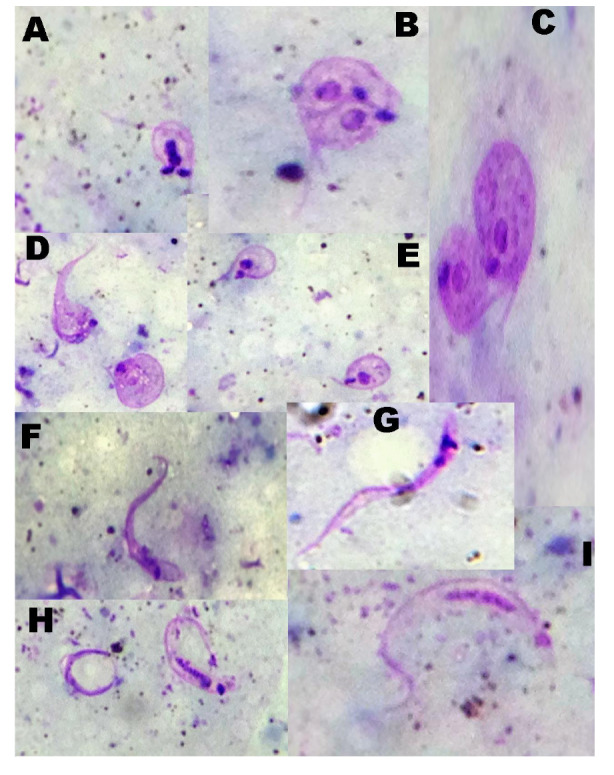
Formas evolutivas de *Trypanosoma cruzi* M/HOM/VE/09/P6 en contenidos intestinales y en heces u orina de especímenes de *Belminus ferroae*. **A**, **B**. Formas de división. **C**, **D**, **E**. Formas de transición. **F**, **G**. Epimastigotes. **H**, **I**. Tripomastigotes metacíclicos (tinción de Giemsa; 1.000X)

En el cuadro 1 se presenta el número de especímenes de *B. ferroae* analizados, muertos e infectados durante el xenodiagnóstico. En el cuadro 2 se registra la carga parasitaria de los contenidos intestinales, las heces o la orina de especímenes de *B. ferroae*. En el cuadro 3 se cuantifican las diferentes formas evolutivas de *T. cruzi* en *B. ferraoe.* En la figura 1 se pueden observar todos los estadios evolutivos del parásito (epimastigotes, formas de transición, formas de división y tripomastigotes metacíclicos), encontrados dentro del tubo digestivo de *B. ferroae* en los diferentes momentos después de la infección.

**Cuadro 1:** Número de especímenes de *Belminus ferroae* analizados, muertos e infectados durante el xenodiagnóstico

**Especímenes**	**Días después de la infección**						**Total**	**%**
	**10**	**20**	**30**	**40**	**50**	**60**		
Infectados	0/3	0/3	1/3	1/2	1/2	0/2	3	15
No infectados	3/3	3/3	2/3	1/2	1/2	2/2	12	60
Muertos*	0	0	0	0	2	3	5	25
Total diario**	3	3	3	2	4	5	20	100

* Solo incluye los insectos fenecidos espontáneamente.

** Incluye los insectos analizados o muertos espontáneamente.

**Cuadro 2:** Carga parasitaria (tripanosomas/mm^3^) detectada en contenidos intestinales y heces u orina de especímenes de *Belminus ferroae* después de su alimentación en ratones infectados con la cepa M/HOM/VE/09/P6

**Días después de la infección**	**Compresión abdominal**	**Macerado de tubo digestivo**
10	0	0
20	0	0
30	1,62 x 10^4^	1,62 x 10^5^
40	1,90 x 10^3^	4,75 x 10^5^
50	9,5 x 10^3^	9,5 x 10^3^
60	0	0

**Cuadro 3:** Cuantificación de formas evolutivas de *Trypanosoma cruzi*, cepa M/HOM/VE/09/P6, observadas en contenidos intestinales y en heces u orina de especímenes de *Belminus ferroae* alimentados con ratones infectados

**Tiempo después de la infección (días)/formas evolutivas**	**n (%)**
**30**	
Epimastigotes	5 (8,48)
Tripomastigotes	4 (6,78)
Formas de transición	46 (77,97)
Formas de división	4 (6,78)
**Total**	59 (100)
**50**	
Epimastigotes	1 (3,57)
Tripomastigotes	1 (3,57)
Formas de transición	25 (89,29)
Formas de división	1 (3,57)
Total	28 (100)

**Figura 2:**
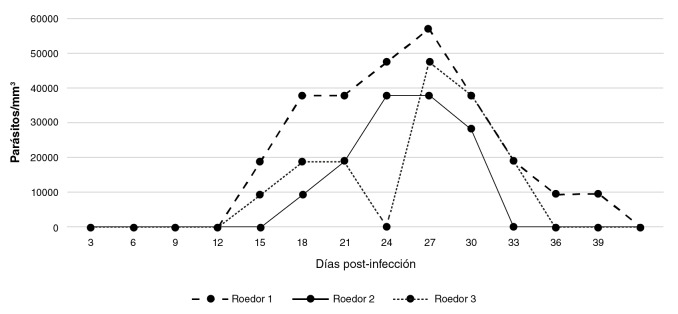
Parasitemias observadas en tres ratones inoculados con macerados de contenidos intestinales de especímenes de *Belminus ferroae* infectados con la cepa (M/HOM/VE/09/P6) de *Trypanosoma cruzi*

En la figura 2 se registra la parasitemia alcanzada por tres ratones albinos inoculados con los macerados del tubo digestivo de los especímenes de *B. ferroae* infectados con la cepa de *T. cruzi* (M/HOM/VE/09/P6).

## Discusión

Los resultados obtenidos demuestran por primera vez la metaciclogénesis de *T. cruzi* en una especie del género *Belminus.* En el contexto epidemiológico y evolutivo de la subfamilia Triatominae; esto reviste interés dado que *B. ferroae* es el único triatomino que logra sustentar su ciclo biológico exclusivamente con huéspedes artrópodos (6,12). A pesar de este comportamiento, la especie no se mostró resistente a la infección por *T. cruzi*.

En futuros estudios, sería interesante evaluar el papel de los agentes tripanolíticos presentes en la hemolinfa de los artrópodos que le sirven de huésped a *B. ferroae*. Ello porque, por ejemplo, recientemente se ha detectado *in vitro* la actividad anti-*T. cruzi* y anti-*T. rangeli* en varias especies de triatominos de importancia sanitaria (19). Por otra parte, en especies de blatodeos, como *Blaberus discoidalis* o *Periplaneta americana*, se han aislado péptidos o lectinas con actividad antibacteriana (por ejemplo, *Escherichia coli*) (21) que podrían exhibir actividad anti-*T. cruzi*.

Asimismo, los resultados del estudio permiten señalar que, eventualmente, *B. ferroae* podría participar en el ciclo de transmisión de *T. cruzi* y representar un riesgo para los humanos en las áreas donde hay poblaciones con tendencias a colonizar el domicilio. Esto se ha corroborado con los porcentajes de infección exhibidos por la especie en condiciones de laboratorio y con diferentes cepas del parásito: cepa Y (50%), y cepa IRHO/CO/98/MTA (33%) (15) y, en el presente estudio, con la cepa M/HOM/VE/09/P6 (15%), así como por los porcentajes de metaciclogénesis detectados: 34 a 38% para la cepa IRHO/CO/98/MTA, 18 a 77% para la cepa Y (15), y 3,5 a 6,78% para la cepa M/HOM/VE/09/P6.

Por otra parte, tanto en la naturaleza como en condiciones de laboratorio, *B. ferroae* ha mostrado preferencia por huéspedes mamíferos (perros, roedores y humanos) y exhibe comportamientos como la cleptohematofagia y el canibalismo, lo que podría contribuir a la adquisición del parásito a partir de otras especies de triatominos o de sus conespecíficos (6,11).

No obstante, aún se requieren estudios adicionales que incluyan la estimación de patrones de defecación y alimentación, lo que, sin duda, permitirá evaluar con mayor precisión el potencial vectorial de la especie.

Se concluyó que el parásito *T. cruzi* logra desarrollar la metaciclogénesis en la especie *B. ferroae* y que los tripomastigotes metacíclicos presentes en sus contenidos intestinales son infectivos para huéspedes vertebrados, resultados de significativo impacto epidemiológico en aquellas áreas geográficas donde esta especie se distribuye naturalmente.
